# Food preferences and associated factors of children aged 7–12 living in temporary shelters after the 2023 earthquake in Turkey: A cross-sectional study

**DOI:** 10.1097/MD.0000000000046321

**Published:** 2026-05-12

**Authors:** Erhan Berk, Hacer Alataş, Nurgül Arslan

**Affiliations:** a Department of Pediatrics, Faculty of Medicine, Malatya Turgut Ozal University, Malatya, Turkey; b Department of Nutrition and Dietetics, Faculty of Health Sciences, Malatya Turgut Ozal University, Malatya, Turkey; c Department of Nutrition and Dietetics, Atatürk Faculty of Health Sciences, Dicle University, Diyarbakir, Turkey.

**Keywords:** children, earthquake, food security, malnutrition, natural disaster

## Abstract

Following an earthquake, there is a probability of disruption in food security that may deteriorate the nutritional status of individuals, particularly children and adolescents residing in the impacted region. This study aimed to assess the nutritional status and associated factors of children aged 7 to 12 residing in temporary shelters in Malatya province, an area significantly impacted by a major earthquake. This study included 317 children aged 7 to 12 years residing in temporary shelters affected by the earthquake. A quantitative method was employed to evaluate nutritional status and its related malnutrition factors, whereas a qualitative approach was used to explore, triangulate, and gather comprehensive information concerning food safety, water hygiene, sanitation, and health in the post-earthquake context. The mean age of the children was 9.42 ± 1.98 years. They were living in temporary shelters for 8.97 ± 1.24 months. Individuals had mild food insecurity due to the earthquakes. Following the earthquake, there was a notable reduction in the consumption of fruits, vegetables, meat, fish, and dairy products (*P* < .05). In the study area, 35.64% were underweight, 21.76% were stunted, and 17.35% exhibited very low weight for height. Gender, family size, father’s employment status, average monthly income, parental education levels, and duration of residence in temporary shelters significantly influenced the body weight of underweight children (*P* < .05). It shows that in order to ensure food security after a major disaster, socioeconomic conditions should be improved, assistance for adequate and balanced nutrition for children should be increased, and occupancy rates of emergency shelters should be limited.

## 1. Introduction

On February 6, 2023, Kahramanmaras in Southern Turkey experienced earthquakes of magnitudes 7.7 and 7.6, impacting 11 cities and approximately 15 million individuals. The World Health Organization (WHO) classified the situation as a Grade 3 emergency, requiring a significant response due to the breakdown of the healthcare delivery system and interruptions in the medical supply chain.

The threat of food insecurity following natural disasters arises not only from the destruction of food reserves and systems, but also from the impairment of individuals’ livelihoods and assets.^[1]^ The repercussions of natural disasters, specifically earthquakes, floods, and droughts, can endure for extended periods. Damage to infrastructure that affects individuals’ livelihoods, including land, agricultural inputs, livestock, and roads, compromises food security.^[2]^

Studies suggest that malnutrition rates tend to increase in the aftermath of natural disasters, particularly earthquakes. For instance, increased child malnutrition has been observed following the Wenchuan earthquake in China, a tsunami in Sri Lanka, and severe flooding in India.^[3–5]^ In Kang County, China, the prevalence of underweight, stunting, and wasting among children rose from 0 to 5.9%, 6.6 to 10.8%, and 1.3 to 4.0%, respectively, within 2 years post-earthquake.^[4]^ Similarly, in India, which borders Nepal, children living in flood-affected households showed a significantly higher likelihood of being underweight (adjusted prevalence ratio 1.86; 95% CI 1.04–2.30) and stunted (adjusted prevalence ratio 1.60; 95% CI 1.05–2.44) compared to those in unaffected households.^[5]^ Following the earthquake in Nepal, there may have been an elevated risk of malnutrition among children, akin to the consequences of these natural disasters.

A 2015 study in Kaski, Nepal, involving children aged 5 to 10 years, found that 44.2% were stunted, 12.3% were wasted, and 35.4% were underweight. The study additionally established a correlation between underweight and family occupation, as well as the family’s economic status, while wasting was associated with the mother’s educational status.^[6]^

Malatya is one of the most severely affected cities worldwide. Critical aspects, including food security and the water supply system, were partially or completely disrupted. Such food shortages can contribute to severe protein-energy malnutrition and other nutritional deficiencies, further increasing the risk of disease and mortality.^[7]^

Although many studies have examined the nutritional status of children under 5 years of age, research focusing on school-aged adolescents remains limited. Limited research has explored the nutritional status of school-aged adolescents following natural disasters such as earthquakes. Following an earthquake, there is a probability of disruption in food security that may deteriorate the nutritional status of individuals, particularly children and adolescents residing in the impacted region. Moreover, eating disorders may also arise as a consequence of post-traumatic stress disorder. Therefore, to mitigate malnutrition, it is essential to identify factors that influence nutritional status. This study aimed to assess the nutritional status and associated factors of children aged 7 to 12 years residing in temporary shelters in Malatya province, which was impacted by a significant earthquake. This study aimed to evaluate the nutritional status and influencing factors among children aged 7 to 12 years living in temporary shelters in Malatya province, which was severely affected by the earthquake.

## 2. Materials and methods

### 2.1. Study population and design

A cross-sectional, community-based study was conducted among children living in temporary shelters in Malatya Province, which was affected by the earthquake in Turkey. The study included 317 children aged 7 to 12 years residing in temporary shelters impacted by the earthquake. This study was conducted in accordance with the principles outlined in the 2015 Helsinki Declaration. This study was approved by the Türkiye Disaster and Emergency Management Authority, the governor’s office, and the Bingöl University Ethics Committee on May 30, 2023 (E-33117789-044-109911).

This study employed a mixed-method approach, incorporating both quantitative and qualitative techniques. A quantitative method was employed to evaluate nutritional status and its related malnutrition factors, whereas a qualitative approach was used to explore, triangulate, and gather comprehensive information concerning food safety, water hygiene, sanitation, and health in the post-earthquake context.

Children residing in containers were chosen randomly. The study included mothers with children aged 7 to 12 in selected containers to gather information regarding their children’s nutritional status through both quantitative and qualitative methods. Children aged 7 to 12 were likewise incorporated in the anthropometric measurements within the designated containers.

### 2.2. Data collection

A structured questionnaire was used to conduct face-to-face interviews with the child’s mother to gather quantitative data. The questionnaire encompassed the participants’ age, educational attainment, employment status, duration of residence in temporary shelters, health conditions, and other factors. The survey questions were taken from a similar study to evaluate nutritional status after the Nepal earthquake.^[8]^

Children’s nutritional status was evaluated by examining their meal patterns and the frequency of food consumption in detail. The food consumption frequency questionnaire comprises 56 items. For food consumption frequency, all food groups (milk and dairy products, eggs, meat and meat products, legumes, cereals, fruits and vegetables, oils, desserts, and beverages) were questioned before and after the earthquake. Children were questioned about the frequency of their food consumption, which was categorized as every meal, daily, 1 to 3 times per week, 4 to 5 times per week, once every 15 days, once a month, or never. Consumption frequency was classified as “little” for intake every 15 days or once a month, “a lot” for daily, every meal, 1 to 3 times per week, and 4 to 5 times per week, while “never” was assigned to complete absence of consumption.

### 2.3. Anthropometric measurement

Anthropometric measurements, in accordance with WHO guidelines,^[9]^ were obtained using the Seca digital weighing scale and a stature meter. The weighing scale was calibrated daily using standard weights. The Seca Digital Scale exhibited a precision of 0.1 kg. Height was measured to the nearest 0.1 cm. Children were directed to stand on the balance wearing lightweight clothing, without footwear, with their feet positioned apart and gazing straight ahead. Height was measured without footwear or headgear. The child’s age was determined by subtracting the date of birth from the interview date. Mothers were asked to recall the height and weight of their children before the earthquake. Children’s height and weight prior to the earthquake were documented based on the mothers’ accounts.

### 2.4. Nutritional status

The nutritional status of children was evaluated based on weight-for-age (WAZ), height-for-age (HAZ), and weight-for-height (WHZ) according to the WHO guidelines. Malnutrition assessment was performed using age-specific weight and height measurements, covering 3 key indicators: underweight, stunting, and wasting.

Underweight: Children whose WAZ was more than 2 standard deviations below (−2SD) the median reference population were classified as underweight. The classification was divided into underweight (< −2SD) and not underweight (> −2SD).

Stunting: Those whose HAZ was below −2SD from the median reference were considered stunted. The classification was made as stunted (< −2SD) and non-stunted (> −2SD).

Wasting: Children whose WHZ ratio was below −2SD from the median reference were categorized as wasted. This was further classified as wasted (< −2SD) or not wasted (> −2SD).

### 2.5. Food security

Food insecurity is defined as the absence of access to sufficient food or the lack of exposure to any of the 3 most critical conditions: depletion of food supplies, going to bed hungry, or experiencing an entire day or night without food.^[10]^

*Mild food insecurity:* Defined by periodic or frequent limitations in food availability and a diet lacking variety, yet without experiencing the most severe conditions such as food depletion, going to bed hungry, or spending an entire day or night without nourishment.

*Moderate food insecurity:* Defined by sporadic or frequent reliance on a repetitive or unappealing diet, occasionally or infrequently reducing meal portions or frequency, yet without facing the most critical conditions such as food depletion, going to bed hungry, or enduring a full day and night without eating.

*Severe food insecurity:* having encountered at least one of the following conditions within the past 12 months: exhaustion of food supplies, going to bed hungry, or spending an entire day or night without food.

### 2.6. Statistical analyses

Statistical analyses were conducted using the SPSS software (version 22.0; SPSS Inc, Chicago). The Shapiro–Wilk test was used to assess data normality, and any data deviating from a normal distribution underwent natural logarithmic transformation. Descriptive statistics were utilized to provide a summary of the background characteristics of the study population, presenting means and standard deviations for continuous data and proportions for categorical variables. The chi-square (χ²) test was applied to evaluate the independence of categorical variables, while analysis of variance with the Scheffe post hoc test was employed to examine differences among nutritional status groups.

### 2.7. Ethics approval

This study was approved by the Ethics Committee of Bingöl University (code E 33117789-044-109911) in accordance with the principles of the Declaration of Helsinki. Informed consent was obtained from the families and children who voluntarily participated in this study.

## 3. Results

The average age of the children participating in this study was 9.42 ± 1.98 years. Children from families comprising 4 to 6 members represented 37.54%. The educational attainment of fathers is predominantly secondary school, whereas that of mothers is primarily primary school. Most fathers were employed and insured, while most mothers were homemakers (Table 1).

**Table 1:** Background related characteristics of study population.

Age of the child (yr)	X̄±S.D	%
	9.42 ± 1.98	
	n	
Sex		
Male	169	53.31
Female	148	46.69
Number of family members		
1–3 individuals	110	34.70
4–6 individuals	119	37.54
6 individuals and above	88	27.76
Number of siblings		
1–2 siblings	75	23.66
3–5 siblings	185	58.36
5 siblings and above	57	17.98
Education of child’s father		
Illiterate	10	3.15
Primary School	88	27.76
Secondary School	125	39.43
High School	81	25.55
University	10	3.15
Master degree-PhD	3	0.95
Education of child’s mother		
Illiterate	24	7.57
Primary School	195	61.51
Secondary School	54	17.03
High School	38	11.99
University	5	1.58
Master degree-PhD	1	0.32
Occupation of child’s father		
Not working	42	13.25
Retired	10	3.15
Civil servant	21	6.62
Insured worker	181	57.10
Uninsured worker	23	7.26
Self-employed	38	11.99
Occupation of child’s mother		
Housewife	254	80.13
Retired	21	6.62
Civil servant	5	1.58
Uninsured worker	32	10.09
Self-employed	5	1.58

Most children residing in temporary shelters are accompanied by family members. Tap water is the predominant source of drinking water for children. The majority of children used squat toilets. Forty-eight percent of children did not utilize soap or water for handwashing. Most children had acquired illnesses in these shelters. Most of the children experienced influenza (Table 2).

**Table 2:** Household water- and sanitation-related characteristics of the study population.

How long have you been living in temporary shelters? (mo)	X̄±S.D	%
	8.97 ± 1.24	
	n	
Who do you live with?		
With family	256	80.76
Other (specify)	61	19.24
Main source of drinking water		
Truck water	178	56.15
Household water with purification	45	14.20
Natural fountain water	21	6.62
Pond/river water	18	5.68
Prepared water	55	17.35
Toilet facilities		
Communal area		
Turkish	280	88.33
European	37	11.67
Toilet in the house		
Turkish	207	65.30
European	110	34.70
Hand washing practice		
Before eating	58	18.30
After eating	77	24.29
After defecation	114	35.96
Hand washing materials		
Water only	15	4.73
Soap and water	149	47.00
Ever	153	48.26
Did you get sick during your time in temporary shelters?		
Yes	198	62.46
No	119	37.54
Passed illness		
Diarrhea	88	27.76
Flu	112	35.33
Poisoning	11	3.47
Bronchitis	54	17.03
Allergic disease	4	1.26
Other	47	14.83

Table 3 outlines the characteristics of the study population in relation to household food insecurity. A significant proportion of the individuals (42.27%) reported that they occasionally or frequently lacked sufficient food or had a monotonous diet. They incurred debt (45.74%) or obtained assistance (40.69%) to address food insecurity. Ninety-five percent of the food shortages were attributed to the earthquake. Individuals predominantly prepared their breakfast, lunch, and dinner in containers.

**Table 3:** Household food insecurity-related characteristics of study population.

Food insecurity	n	%
Food shortage	55	17.35
Going to bed hungry (not having food)	124	39.12
Going all day and night without eating	26	8.20
Sometimes or often not having enough food or a more monotonous diet than desired	134	42.27
Strategies to cope with food insecurity		
Borrowing	145	45.74
Help	129	40.69
Other	117	36.91
Reason for food shortage		
Earthquake	288	90.85
Financial problem	96	30.28
Not available	75	23.66
Other	43	13.56
How do you provide for your meals		
Breakfast		
Preparing our own containers	291	91.80
Getting from soup kitchens	26	8.20
Lunch		
Preparing our own containers	285	89.91
Getting from soup kitchens	32	10.09
Dinner		
Preparing our own containers	296	93.38
Getting from soup kitchens	21	6.62

Most individuals had 2 meals before and after the earthquake. A significant proportion of the respondents reported skipping meals (Table 4).

**Table 4:** Nutritional habits of individuals before and after the earthquake.

	Before the earthquake		After the earthquake		*P*
	n	(%)	n	(%)	
Main meal					
1 meal	56	17.67	95	29.97	.206
2 meals	195	61.51	145	45.74	
3 meals	66	20.82	77	24.29	
Snack					
1 meal	75	23.66	16	5.05	.517
2 meals	101	31.86	35	11.04	
Do you skip meals?					
Yes	251	79.18	240	75.71	.149
No	66	20.82	77	24.29	

**Figure 1.**
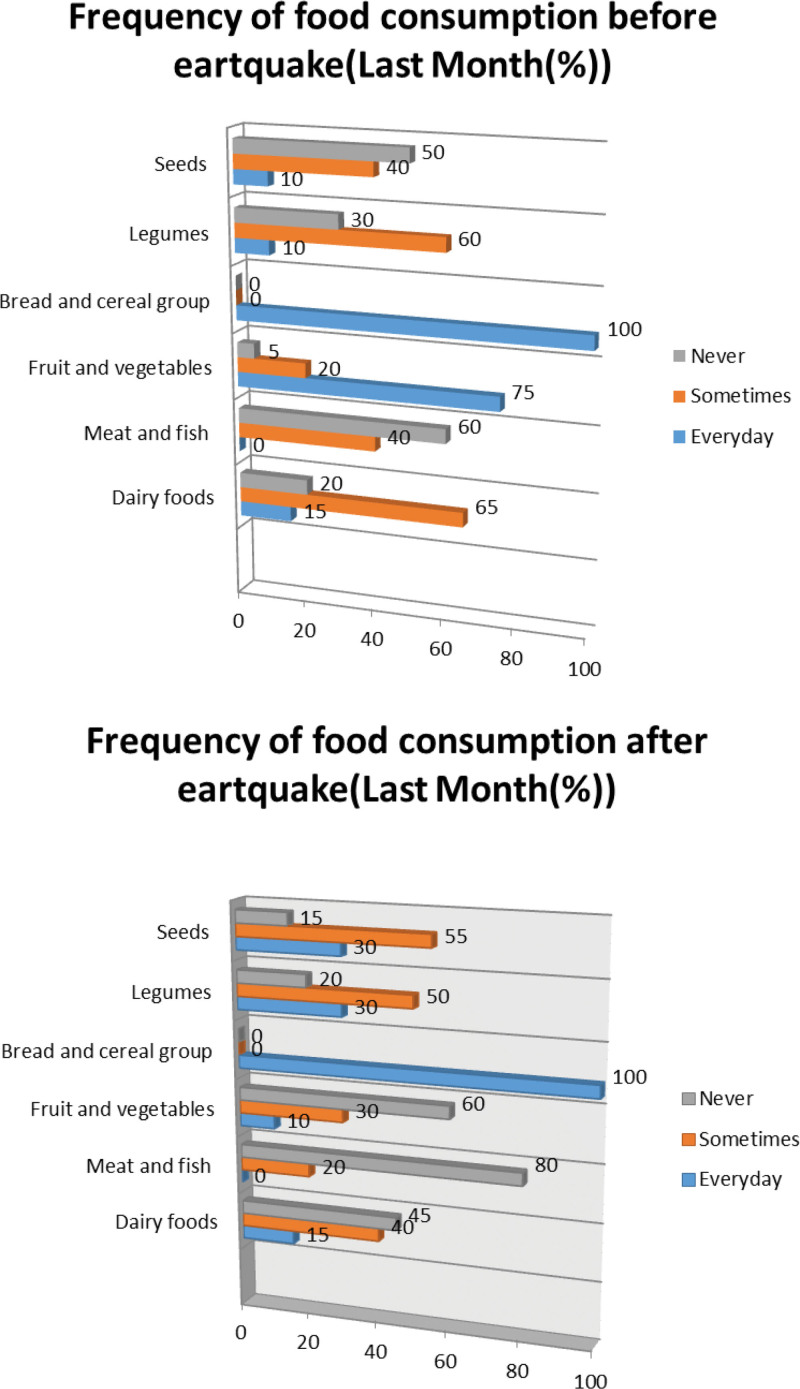
Depicts the changes in the frequency of food consumption among individuals before and after the earthquake. It was established that individuals consumed oilseeds 10% of the time prior to the earthquake and 30% daily thereafter, fruits and vegetables 75% of the time before the earthquake and 10% daily afterward, meat and fish 40% occasionally before the earthquake and 20% occasionally after, and refrained from consuming dairy products 45% of the time following the earthquake.

Figure 1 depicts the changes in the frequency of food consumption among individuals before and after the earthquake. It was established that individuals consumed oilseeds 10% of the time prior to the earthquake and 30% daily thereafter, fruits and vegetables 75% of the time before the earthquake and 10% daily afterward, meat and fish 40% occasionally before the earthquake and 20% occasionally after, and refrained from consuming dairy products 45% of the time following the earthquake.

Table 5 presents body weight and height measurements of individuals, categorized by gender, before and after the earthquake. A significant increase in height was noted among girls (*P* < .05).

**Table 5:** Body weight and height measurements by gender before and after the earthquake.

	Girls	*P*	Boys	*P*
	(X̄±SD)		(X̄±SD)	
Body weight (kg) (Before earthquake)	26.25 ± 6.48	.537	29.35 ± 11.45	.087
Body weight (kg) (After earthquake)	27.50 ± 5.25		28.15 ± 7.18	
Height (cm) (Before earthquake)	133.01 ± 14.78	.007	134.21 ± 13.99	.279
Height (cm) (After earthquake)	136.09 ± 16.45		138.49 ± 15.19	

Table 6 presents the nutritional status of the 237 malnourished children who participated in the study alongside their parents. In the seventh and eighth months following the severe earthquake, approximately one-third of the children (35.64%) in the study area were underweight, 21.76% were stunted, and 17.35% exhibited very low WHZ. The mean Z-score for WAZ (underweight) was −1.51 (95% CI: −1.733 to −1.489), while the mean Z-score for HAZ (stunting) stood at −2.15 (95% CI: −2.246 to −2.329). Additionally, the mean Z-score for WHZ (wasting) was recorded as −0.28 (95% CI: −0.312 to −0.459).

**Table 6:** Examination of malnutrition in children with Z score.

	n	(95% CI)	%
*Normal weight* Mean Z-score for weight for age (95% CI)	80	1.15 (1.279 to 1.455)	25.24
*Underweight* Mean Z-score for weight for age (95% CI)	113	−1.51 (−1.733 to −1.489)	35.65
*Stunting* Mean Z-score for height for age (95% CI)	69	−2.15 (−2.246 to −2.329)	21.76
*Wasting* Mean Z-score for weight for height (95% CI)	55	−0.28 (−0.312 to −0.459)	17.35

Multiple regression analysis was performed to determine the demographic factors influencing body weight in underweight children (*F* = 29.033, *P* < .001). All variables collectively accounted for 64% of the children’s body weight. Gender, family size, father’s employment status, average monthly income, parental education levels, and duration of residence in temporary shelters significantly influenced body weight in underweight children (*P* < .05). The order of predictive importance for the total score was as follows: gender, family size, fathers’ employment status, and duration of residence in temporary shelters (Table 7).

**Table 7:** Multiple regression analysis of demographic characteristics affecting body weight of children with low body weight.

Features	B	Std error	Beta		*P*	95.0% CI	
						Lower bound	Upper bound
Gender							
Female (ref.)							
Male	−6.037	1.221	−.308	−5.408	**.000**	−8.0179	−4.127
Number of family members							
3–6 people (ref.)							
6 people and above	−5.139	1.105	−.165	−4.672	**.000**	−7.027	−3.146
Father’s employment status							
Yes (ref.)							
No	−2.216	2.314	−.092	−1.912	.321	−6.127	2.329
Average monthly family income							
As much as minimum wage (ref.)							
Below minimum wage	−2.277	1.019	−.066	−2.104	**.031**	−4.503	−1.315
Father’s education status							
Literate (ref.)							
Illiterate	−6.694	1.107	−.638	−5.258	**.781**	−8.179	−4.473
Mother’s education status							
Literate (ref.)							
Illiterate	−6.178	1.101	−.398	−5.441	**.479**	−8.215	−4.441
Life expectancy in temporary shelters							
3–6 months (ref.)							
6–9 months	−6.167	1.107	−.448	−5.516	**.001**	−5.806	−4.13

*F*: 29.033, *R*: 0.802, *R*^2^: 0.643, Std. error of the estimate: 14.233. Dependent variable: body weight. Bold values indicate statistically significant (*P* > .001).

CI = confidence interval.

## 4. Discussion

This study highlights that food security was significantly compromised in the aftermath of the disaster. Families have lost their homes and sources of livelihood, forcing them into temporary shelters, which in turn has triggered substantial socioeconomic and cultural changes. These shifts directly contributed to heightened food insecurity. A considerable proportion of participants reported experiencing food shortages as a result of the earthquake, relying heavily on humanitarian aid, or incurring debt to secure food. While the majority of households prepared their own meals, dietary diversity was markedly reduced, with many individuals subsisting on monotonous diets lacking essential nutrients. Similar findings have been reported in post-disaster contexts where food insecurity and reduced dietary variety have been widely documented.^[5,11,12]^

In terms of children’s nutrition, the present study found no significant changes in meal frequency before and after the earthquake; however, the types of food consumed shifted considerably. The consumption of fruits, vegetables, meat, fish, and dairy products has declined, reflecting both reduced availability and diminished household purchasing power. Such dietary shifts are particularly concerning for children, as reliance on carbohydrate-dense, nutrient-poor foods may impair growth, compromise immune function, and elevate the risk of neurodevelopmental delays associated with chronic undernutrition.^[13,14]^ These observations reinforce the importance of ensuring dietary diversity and targeted nutritional support in post-disaster interventions to safeguard children’s health and development.

The current study observed no difference in meal frequency among children before and after the earthquake; however, the frequency of food consumption was altered. The consumption of fruits, vegetables, meat, fish, and dairy products has declined. This reduction poses a risk to the growth and development of children, particularly for those on a carbohydrate-heavy diet, potentially increasing the likelihood of neurodevelopmental issues caused by malnutrition.

This study is the first to demonstrate that outdoor conditions are associated with insufficient dietary intake across a majority of food groups. Following the Great East Japan Earthquake, a cohort study reported that changes in living conditions and unemployment were inversely correlated with consumption of fruits, vegetables, and soy-based products.^[15]^ Similarly, a preliminary investigation of survivors from Iwate Prefecture indicated that better living conditions in the year following the disaster were associated with healthier dietary habits, including increased consumption of soy products, fish and seafood, fresh fruits and vegetables, and dairy items.^[16]^ A cross-sectional study conducted in 4 urban areas of France revealed that immigrant status was significantly associated with a lower frequency of fruit and vegetable consumption (<3.5 times daily) and dairy product intake (<2 times daily).^[17]^ Furthermore, individuals suffering from severe malnutrition are more likely to consume fruits and vegetables, meat, seafood, and eggs less than once per day, along with infrequent intake of dairy products. Limited monthly food budget, temporary shelter accommodation, and absence of household income were also associated with reduced seafood consumption.^[17]^

In addition, experimental studies have highlighted that exposure to natural environments can promote healthier food choices; for example, walking in nature or even viewing natural images encourages healthier snack selection compared to urban settings.^[18]^ Socioeconomic factors also been shown to play a decisive role in diet quality, especially among low-income and immigrant populations. The French ABENA study demonstrated that immigrant status was associated with a lower intake of fruits, vegetables, and dairy products, while severe food insecurity was linked to very low consumption of fruits, vegetables, meat, seafood, and eggs. Moreover, a reduced monthly food budget, temporary housing, and lack of income were strongly associated with decreased seafood intake.^[19]^ Similarly, a study among migrants in Morocco found that homelessness adjusted odds ratio (AOR) = 6.32, lack of social support (AOR = 2.30), and low monthly income (AOR = 8.21) were strongly correlated with poor dietary diversity.^[20]^ Additionally, research suggests that poor dietary habits in low-income groups emerge from complex systemic interactions, including accessibility, affordability, cultural factors, and individual preferences, indicating that economic interventions alone may be insufficient and that systems-based approaches are crucial for effective policy development.^[21]^

The findings from this study indicated that the occurrence of undernutrition, particularly underweight, was higher than that of normal weight. Comparable results have been documented in other investigations that assessed the prevalence of undernutrition.^[22–24]^ However, numerous studies from different nations have reported a greater prevalence of undernutrition-related stunting than that observed in the current study.^[25,26]^ These disparities in undernutrition prevalence may stem from variations in nutrient intake, dietary diversity, socioeconomic status, environmental exposures, and cultural practices rather than genetic potential for optimal height attainment.^[27]^ Context-specific determinants, such as poverty, food insecurity, maternal education, inadequate water, sanitation, and hygiene conditions have been repeatedly identified as critical risk factors influencing the prevalence of both underweight and stunting in children.^[28,29]^

The findings from this study indicated that the occurrence of undernutrition, particularly underweight, was higher than that of normal weight. Comparable results have been documented in other investigations assessing the prevalence of undernutrition^[30–33]^; however, numerous studies from different nations indicated a greater prevalence of undernutrition-related stunting than that observed in the current study.^[6,34]^ The disparities in undernutrition prevalence may stem from variations in nutrient intake and socioeconomic and cultural factors, rather than the genetic potential for optimal height attainment.

In this study, gender, number of family members, fathers’ employment status, and duration of stay in temporary shelters were significantly associated with children’s underweight. Evidence from multiple contexts supports these findings, highlighting the critical role of parental education and socioeconomic status in child nutrition outcomes. Research conducted in western Nepal revealed that children of illiterate mothers were almost twice as likely to suffer from malnutrition compared to those whose mothers were literate.^[35]^ Similarly, a study in the Kaski District of Nepal identified a strong link between maternal education and child stunting, emphasizing education as a protective factor against undernutrition.^[36]^ Findings from urban slums in India also demonstrated a significant association between maternal education and child malnutrition, further reinforcing the maternal education–nutrition nexus across different low-resource settings.^[37]^

Paternal education has also been identified as an important determinant in certain contexts. A study in Nepal reported a significant correlation between paternal education and both underweight and stunting.^[38]^ However, evidence is mixed: a study conducted in central India revealed no association between paternal education and underweight status, whereas male sex and maternal education were strongly linked to underweight prevalence.^[39]^ These findings underscore the multidimensional nature of child malnutrition, where maternal education consistently emerges as a critical determinant, whereas the role of paternal education may vary across cultural and socioeconomic contexts.

A study conducted in Nepal reported no significant relationship between family size and prevalence of underweight, stunting, or wasting among children.^[38]^ In contrast, research from other countries has demonstrated a positive association between larger family size and child malnutrition. For instance, studies from Egypt,^[40]^ Ethiopia,^[41]^ and India^[37,39]^ have consistently documented that children from larger households are more likely to experience malnutrition. These findings suggest that increased household size may limit food and healthcare resources, reduce per capita food availability, and compromise childcare quality.

The discrepancy between the current study and evidence from other regions may be explained by contextual differences. In post-disaster settings such as the environment examined here, the influence of family size on child nutrition may be masked by more immediate determinants, including food insecurity, displacement, and psychosocial stressors. Thus, while family size is a recognized determinant of undernutrition in stable settings, its role may be altered in environments where disasters disrupt household structure and resource distribution.

Household income is widely recognized as a critical determinant of children’s nutritional outcomes. However, the relationship between socioeconomic status and undernutrition is not always consistent across different contexts. For instance, a study conducted in Central India found no significant association between household wealth and the prevalence of underweight, stunting, or wasting among children.^[39]^ In contrast, another Indian study reported that children from low-income families were nearly 3 times more likely to be underweight and stunted compared to those from higher-income households,^[42]^ research from Mysore, India, demonstrated a strong link between low socioeconomic status and the prevalence of underweight in children.^[43]^ Additional investigations from both India and other low- and middle-income countries have reinforced this finding, consistently showing that economic deprivation contributes to poor nutritional status by limiting access to adequate food, healthcare, and sanitation services.^[37,44]^

The inconsistency observed between studies may reflect contextual differences in how wealth translates into improved childhood nutrition. In certain settings, cultural practices, maternal education, and household food allocation patterns may attenuate the benefits of a higher income. Conversely, in resource-constrained environments, low socioeconomic status exacerbates the risk of malnutrition by constraining dietary diversity and access to essential healthcare services. These findings underscore the need to consider income as an important, yet context-dependent determinant of undernutrition in children.

Unlike findings from earlier studies, the present research indicates that the socioeconomic determinants of child nutrition were strongly shaped by the extensive loss of household assets and adverse living conditions resulting from the 2023 earthquake. Natural disasters often disrupt livelihoods, reduce income-generating opportunities, and deplete household resources, exacerbating food insecurity and limiting access to adequate nutrition.^[44]^ In this context, factors such as parental education or family income, commonly associated with child nutrition under stable conditions, may lose their protective effects when families are displaced, unemployed, or forced to rely on temporary shelters. Previous disaster-related research has similarly shown that post-disaster environments magnify the effects of poverty and resource scarcity, increasing children’s vulnerability to undernutrition.^[5,45]^ These findings suggest that, in disaster-affected populations, structural losses and environmental hardships may outweigh traditional socioeconomic predictors, highlighting the need for targeted nutritional interventions and recovery policies tailored to post-disaster settings.

### 4.1. Limitations of study

This study had some limitations that need to be addressed. Initially, there may have been biases against the government among sheltered individuals in the present study. Consequently, they may have responded to the survey questions with a bias. The primary objective of this survey was to ascertain insufficient consumption of key food groups. Subsequently, a detailed food frequency questionnaire was administered. Nevertheless, data regarding portion sizes were not assessed. Consequently, it was not feasible to determine the quantity of each item consumed. Third, under exceptional and atypical social circumstances following a significant disaster, daily dietary consumption may be profoundly influenced by alterations in familial dynamics among evacuees (e.g., death or physical separation). However, this information was not examined in the present study.

## 5. Conclusions

The factors influencing the nutritional intake of children residing in temporary shelters following the Great Earthquake in Southeastern Turkey on February 6, 2023, were thoroughly examined. The results of this study revealed a decline in the consumption of fruits, vegetables, meat, fish, and dairy products among children living in temporary shelters after the earthquake compared to the pre-earthquake period. The incidence of malnutrition in children, particularly underweight children, exceeds that of normal weight children. Sex, family size, paternal employment status, and duration of residence in temporary shelters were significantly correlated with children’s underweight status.

Our research indicates that to guarantee food security following a significant disaster, socioeconomic conditions must be enhanced, assistance should be augmented for sufficient and balanced nutrition, and the occupancy of emergency shelters should be restricted.

We anticipate that governments and authorities will promptly implement the requisite measures to prepare for future disasters.

## Author contributions

**Conceptualization**: Erhan Berk.

**Investigation**: Hacer Alataş.

**Methodology**: Erhan Berk, Hacer Alataş, Nurgül Arslan.

**Project administration**: Erhan Berk, Hacer Alataş.

**Resources**: Erhan Berk, Hacer Alataş.

**Software**: Erhan Berk, Nurgül Arslan.

**Validation**: Hacer Alataş.

**Visualization**: Erhan Berk, Hacer Alataş.

**Writing – original draft**: Erhan Berk, Hacer Alataş, Nurgül Arslan.

**Writing – review & editing**: Erhan Berk, Hacer Alataş, Nurgül Arslan.
